# Characterisation of the *Fusarium graminearum*-Wheat Floral Interaction

**DOI:** 10.4061/2011/626345

**Published:** 2011-10-05

**Authors:** Neil A. Brown, Chris Bass, Thomas K. Baldwin, Huaigu Chen, Fabien Massot, Pierre W. C. Carion, Martin Urban, Allison M. L. van de Meene, Kim E. Hammond-Kosack

**Affiliations:** Department of Plant Pathology and Microbiology, Centre for Sustainable Pest and Disease Management, Rothamsted Research, Harpenden, Hertfordshire AL5 2JQ, UK

## Abstract

Fusarium Ear Blight is a destructive fungal disease of cereals including wheat and can contaminate the crop with various trichothecene mycotoxins. This investigation has produced a new **β**-glucuronidase (GUS) reporter strain that facilitates the quick and easy assessment of plant infection. The constitutively expressed *gpdA:GUS* strain of *Fusarium graminearum* was used to quantify the overall colonisation pattern. Histochemical and biochemical approaches confirmed, in susceptible wheat ear infections, the presence of a substantial phase of symptomless fungal growth. Separate analyses demonstrated that there was a reduction in the quantity of physiologically active hyphae as the wheat ear infection proceeded. A simplified linear system of rachis infection was then utilised to evaluate the expression of several *TRI* genes by RT-qPCR. Fungal gene expression at the advancing front of symptomless infection was compared with the origin of infection in the rachis. This revealed that *TRI* gene expression was maximal at the advancing front and supports the hypothesis that the mycotoxin deoxynivalenol plays a role in inhibiting plant defences in advance of the invading intercellular hyphae. This study has also demonstrated that there are transcriptional differences between the various phases of fungal infection and that these differences are maintained as the infection proceeds.

## 1. Introduction

Fusarium Ear Blight (FEB) disease also referred to as Fusarium head scab (http://www.scabusa.org) is a destructive fungal disease that has the potential to devastate wheat, barley, rye, oat, or maize crops just weeks before harvest. All of the major wheat producing countries (http://faostat.fao.org) have reported serious and repeated FEB outbreaks in the past decade, making the impact of *Fusarium *infections a global issue. This worldwide re-emergence is believed to be driven by changes in climate and agronomic practices. CIMMYT describes FEB as a major limiting factor to wheat production across the world [1]. Over the last 10 years, the average FEB incidence in UK wheat fields was 39% (http://www.cropmonitor.co.uk). *Fusarium graminearum *(teleomorph *Gibberella zeae*) is a filamentous ascomycete and one of the main causal agents of FEB throughout Europe, Asia, and the Americas [1, 2]. Besides dramatically reducing yield, grain quality is affected through the selective loss of albumin and gluten proteins [3]. The grains harvested from an infected crop are also contaminated with various fungal mycotoxins often making them unsuitable and/or unsafe for human consumption, animal feed, or malting purposes [4, 5]. Farmers in the UK pay the mill, where their grain is processed, to test for the presence of the type B trichothecene mycotoxin deoxynivalenol (DON) and determine that the levels are below the EU safety guidelines (http://www.hgca.com) [6]. Approximately one infected ear per square metre of the crop would be sufficient for grain found to contain DON to be rejected.

During anthesis, the anthers naturally split to release pollen, which provides an opening for the pathogen to enter the wheat plant. Anther extrusion also exposes the poorly defended inner surface of the floral brackets, which can be penetrated directly [7]. To initiate the disease cycle, ascospores and asexual conidia are either rain splashed or wind dispersed, onto the floral tissues of the wheat crop [8]. Therefore, epidemics on wheat occur when warm and moist weather conditions coincide with flowering. Infection of the individual florets within each spikelet then occurs [9–11]. Once within the spikelet's rachilla, fungal hyphae advance in the intercellular spaces between live wheat cells. Macroscopically, the wheat ear tissue colonised appears symptomless [12]. In the early stages of infection, this symptomless growth represents the majority of the colonised tissue. Plant cells then die prior to, or at the same time as, the onset of intracellular colonisation by the pathogen, and this plant cell death coincides with the onset of externally visible disease symptoms on the wheat ears [12]. At the later phases of infection, ascogenous hyphae form just below the wheat cell surface layers in preparation for later perithecial formation [13].

*F. graminearum *produces a range of sesquiterpenoid mycotoxins including several type B trichothecenes, such as DON and its acetylated derivatives 15A-DON and 3A-DON, which are required for full virulence on wheat ears [14–16]. DON inhibits protein synthesis in eukaryotes and prevents polypeptide chain initiation or elongation by binding to the 60S ribosomal subunit [17]. The first committed step in the biosynthesis of trichothecenes is the conversion of farnesyl pyrophosphate to trichodiene, which is catalysed by the trichodiene synthase enzyme coded for by the *TRI5 *gene. The role of DON in pathogenicity has been examined via the construction and use of non-DON producing *tri5* gene deficient mutants. The absence of mycotoxin production during wheat ear infection results in an enhanced plant defence response in the form of plant cell wall thickening which impedes rachis colonisation [9]. However, DON has also been implicated in the activation of several wheat defence responses including hydrogen peroxide production and programmed cell death which may aid colonisation [18].

The identification and evaluation of symptomless infection in wheat ears via light and electron microscopy is time consuming and costly [12]. In this study, we created a transgenic *F. graminearum* reporter strain, that expresses the *β*-glucuronidase enzyme (GUS) driven by the constitutive glyceraldehyde 3 phosphate dehydrogenase promoter from *Aspergillus nidulans *(*gpdA*) [19]. This reporter strain enabled the rapid evaluation of symptomless infection throughout the fungal colony in the ear. We then explored the expression of various *Fusarium TRI *genes during the early symptomless and symptomatic phases of wild-type infection by reverse transcriptase—quantitative polymerase chain reaction (RT-qPCR) analysis. This investigation of the infection of a susceptible wheat genotype by *F. graminearum* has (1) correlated the presence of fungal RNA with observed fungal colonisation at the cellular level; (2) compared fungal gene expression at the advancing front of symptomless infection with the origin of infection in the rachis; (3) revealed that the expression of several *TRI* genes is maximal at the advancing front; (4) shown that the centre of the *Fusarium *colony within the wheat ear has only minimal physiologically active hyphae. Overall, this study has shown that there are distinct phases to the *Fusarium* infection within the developing wheat ears and that these phases are maintained as infection proceeds.

## 2. Results

### 2.1. The Transformed Constitutive GUS Expressing Strain Exhibits a Wild-Type Phenotype

Post co-transformation the *in vitro* colorimetric GUS assay confirmed that 38% of the selected hygromycin resistant transformants also contained the *gpdA*:*GUS *reporter construct (data not shown). A transformant which demonstrated a stable high level of GUS activity when grown on potato dextrose agar (PDA) broth was identified by using the quantitative 4-methylumbelliferyl-*β*-D-glucuronide (MUG) assay. Transformant G3 produced 1153 nmol MU min^−1^ mg protein^−1^ StD ± 165 nmol min^−1^ using standard assay conditions. This transformant showed wild-type growth rates on a minimal (SNA) and rich (PDB) media and wild-type asexual and sexual sporulation after growth under near UV/white light on either SNA or carrot agar. Measurement of mean DON concentration by ELISA after growth on DON inducing corn meal agar showed DON production was unaltered (PH-1 (1.6 ppm), G3 (1.5 ppm), analysis of variance (ANOVA), and Fisher *F*-test (*P* > 0.05)).

### 2.2. The Constitutive GUS Strain Shows Wild-Type Pathogenicity

**Figure 1:**
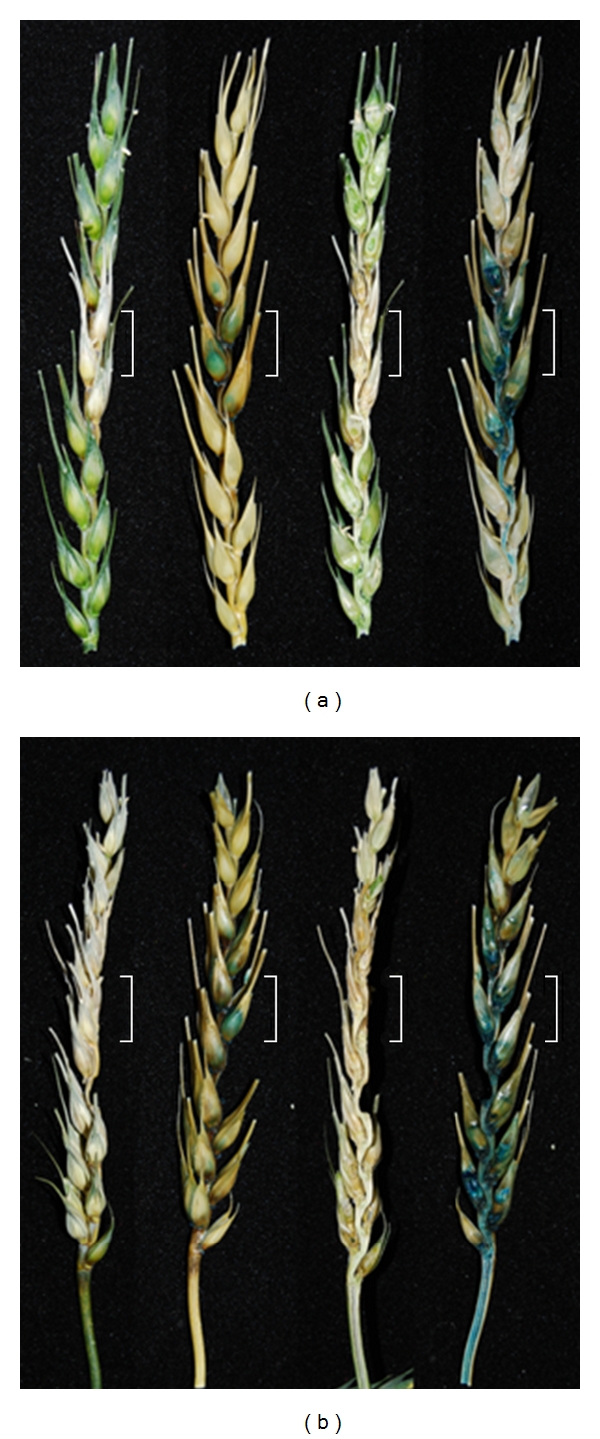
X-gluc stained susceptible Bobwhite wheat ear at 8 days (a) and 12 days (b) post point inoculation with the constitutive GUS expressing strain. The four ears displayed from left to right in each panel were either photographed before and then after staining of the same intact ear or before and after staining of an ear longitudinally sliced through the midpoint. The white brackets surrounding a portion of each ear pair indicates the two spikelets which received the original *F. graminearum* inoculum. Note: Mock (water-only) inoculated ear demonstrated no nonspecific GUS staining (data not shown).

The high levels of GUS produced by the G3 strain allowed the infection process to be followed by dissection and histochemical staining at 8 and 12 days post inoculation (dpi). The specificity of macroscopic GUS staining was confirmed to localise to the fungal hyphae (see Supplementary Figure 1 in Supplementary Material available online at doi: 10.4061/2011/626345). Staining at 8 dpi revealed hyphae had colonised beyond the visible disease symptoms (Figure 1). By 12 dpi, hyphae had grown throughout the entire rachis below the inoculated spikelet and down into the peduncle (Figure 1).

**Figure 2:**
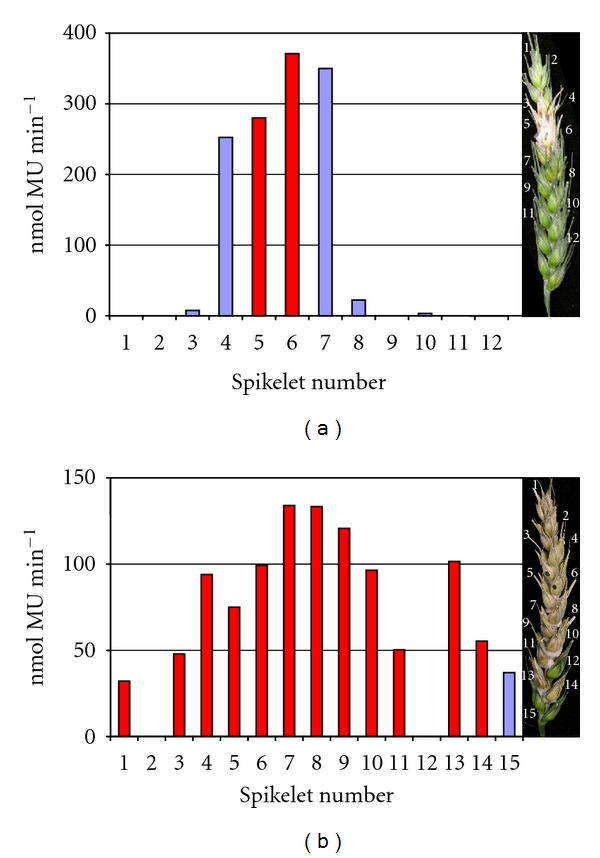
GUS activity (nmol MU minute^−1^) of individual spikelets in representative wheat ears (susceptible cv. Bobwhite) inoculated with the constitutive GUS expressing *Fusarium* strains and harvested at either 8 dpi (a) or at 16 dpi (b). The right portion of each panel is a photograph showing the macroscopic disease symptoms on the ear immediately prior to all the spikelets being sampled. The bars in red represent symptomatic infection, whilst the bars in blue represent symptomless infection. In technical replicates, the values obtained never varied more than 5% from each other.

**Figure 3:**
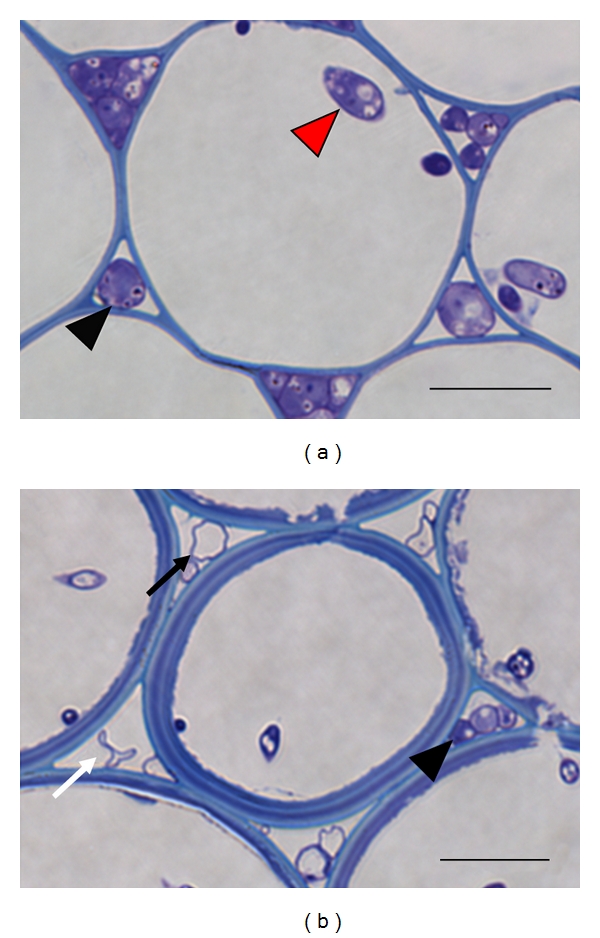
The reduction in fungal biomass at the centre of the colony as infection progressed throughout the wheat ear by 12 dpi. Images of transverse 1 *μ*m LR white sections of cortical cells within various rachis internodes stained with 0.1% TBO pH 9. The first rachis internode at 5 dpi (a) and the third rachis internode at 12 dpi (b). Generic legend: black arrowhead: intercellular hyphae, red arrowhead: intracellular hyphae, black arrow: hyphae devoid of content, white arrow: collapsed hyphae. Bar: 15 *μ*m.

The fluorescent MUG assay is a more sensitive method to quantify fungal infection. The extent of infection varied significantly between individual wheat ears, typically by 2 spikelets by day 8 and 4 spikelets by day 16. Subsequently, representative ears were evaluated separately, and these results are presented. All spikelets on wheat ears were individually assessed at 8 and 16 dpi (Figure 2). Combined with the histochemical staining, substantial symptomless colonisation continually advanced the infection throughout the wheat ear, relative to the progression of visible disease in that ear. The MUG assay also confirmed that some of the severely bleached spikelets towards the tip of the ear late in infection commonly lacked GUS activity and therefore remained noninfected. Interestingly, on occasions individual spikelets on fully disease ears appeared healthy. For example, at 16 dpi, one spikelet (no. 12) in an otherwise heavily diseased ear had developed no macroscopic disease symptoms. The MUG assay confirmed spikelet 12 to be free of fungal infection and demonstrated the reliability of the system to monitor accurately the progression of infection. Disease-free spikelets were identified on several of the inoculated ears and could be located at any position in the ear. It was also noted that overall there was a 15-fold reduction in total GUS activity between 8 and 16 dpi throughout the spikelets of the ear (Table 1). To determine if this was caused by a reduction in active fungal biomass and a redistribution of resources to the actively growing region of the colony as infection progresses, a detailed microscopic analysis of infected ears was carried out. This revealed that hyphae had indeed lost their cell content at the late time point, 12 dpi (Figure 3), which would account for this reduction in GUS activity.

### 2.3. The Identification of Fungal Hyphae at the Cellular Level Correlates with the Detection of *F. graminearum* RNA

**Figure 4:**
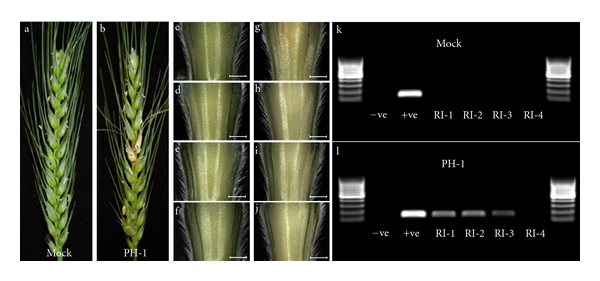
Detection of fungal RNA within the relevant rachis internode correlated with the identification of fungal hyphae at the cellular level (presented in Figure 2). Panels (a) and (b) are photographs of the water-only control (Mock) and the PH-1 infected ears at 5 dpi. The four sequential rachis internodes below the inoculated spikelet from the mock control (c through f) and PH-1 infected (g through j) ears, bar: 1 mm. RT-PCR of the RNA extracted from the rachis internodes of the mock control (k) and the PH-1 infected (l) using primers for *F. graminearum *gamma actin (141 bp) separated on a 2% agarose gel alongside a 100 bp DNA ladder (GeneRuler, Fermentas). RI-1 to RI-4: the sequential rachis internodes below the inoculated spikelet, −ve: the nontemplate negative control, +ve: the *F. graminearum *gDNA positive control. Inoculated spikelets are marked with a black dot.

Colonisation of the rachis represented a simplified linear system for monitoring and quantifying the development of infection which leads to sequential spikelets becoming colonised. Within the rachis internodes, hyphae predominantly grow in one direction, enabling the accurate isolation of the advancing front of infection. The four rachis internodes below the inoculated spikelet were excised at 5 dpi to determine the extent of the macroscopically visible symptoms (Figure 4). The appearance of disease symptoms, in the excised rachis internodes (RI), was also evaluated at the cellular level (Supplementary Figure 2). No hyphae were detected in RI-4, the internode the furthest from the point of inoculation. The active front of infection was identified in RI-3 and consisted of a low number of intercellular hyphae in association with live cortical cells. RI-2 possessed a higher number of intercellular hyphae than RI-3, surrounding live plant cells. The internode closest to the point of inoculation, RI-1, where the rachis infection had begun, contained both inter- and intracellular hyphae. Plant cortical cells and vascular elements in the centre of this rachis internode were intracellularly colonised and dead. However, the infected cell types were surrounded by live sclerenchyma and epidermal cells, accounting for the slight appearance of macroscopically visible disease symptoms in this internode at 5 dpi. 

Total RNA was extracted from 15 pooled rachis internodes (Supplementary Figures 3 and 4), and post DNAase treatment a PCR analysis was done using intergenic primers designed to amplify only genomic DNA (gDNA). These checks confirmed the absence of DNA contamination (Supplementary Figure 4). In the first three rachis internodes below the point of inoculation, fungal RNA was identified via reverse transcription quantitative PCR (RT-PCR) using primers to the housekeeping genes *γ*-actin and *β*-tubulin (see below). This result correlated well with the observation of hyphal infection at the cellular level.

 

### 2.4. *TRI* Gene Expression is Upregulated at the Active Hyphal Front of Symptomless Infection

Five *Fusarium *genes from the *TRI *cluster, namely, *TRI4*,* TRI5*,* TRI6*,* TRI9*, and *TRI14*, along with two housekeeping genes, *γ*-actin and *β*-tubulin, were selected for analysis (Supplementary Table 1). Each *TRI *gene was screened by real-time quantitative PCR (RT-qPCR) for changes in transcript abundance and normalised for fungal biomass within the three colonised rachis internodes at 5 dpi. These *TRI *genes were chosen due to their known involvement in pathogenesis, and mycotoxin production [4, 14, 15, 20–22]. For the 7 dpi, six colonised rachis internodes were sampled from each wheat ear, while the analysis focussed only on the expression of *Tri4*, *Tri5 *and the two housekeeping genes. 

**Figure 5:**
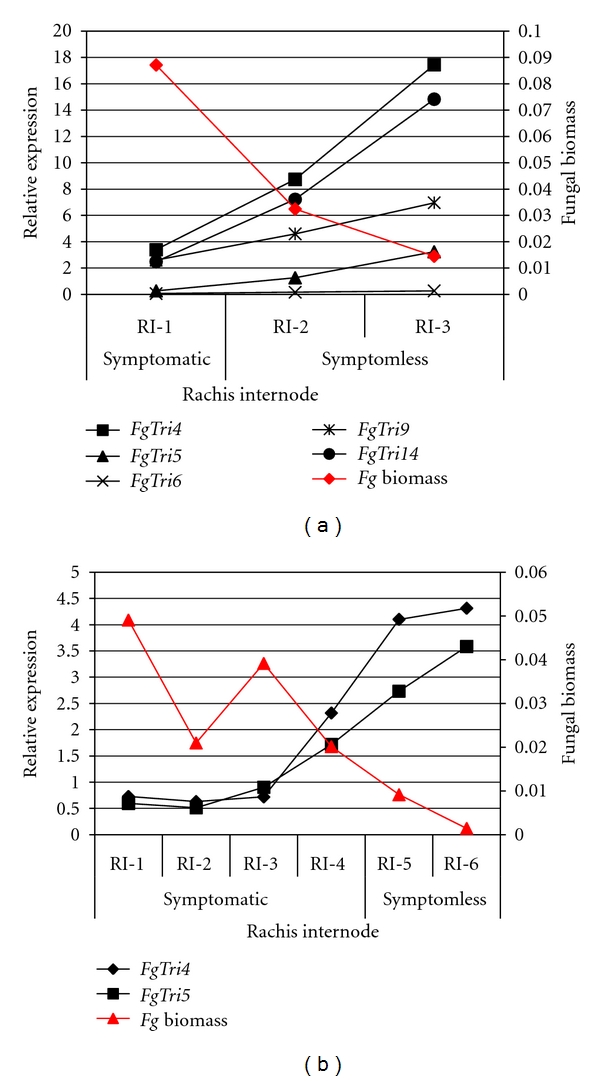
Fungal *TRI *gene expression in the infected rachis internodes in which *F. graminearum *was detected at 5 dpi (a) and 7 dpi (b). Relative expression was measured by RT-qPCR and values normalised for fungal biomass relative to the expression of *F. graminearum γ*-actin and *β*-tubulin. For clarity, the results of the statistical analysis for each gene at 7 dpi are presented in Supplementary Figure 5.

The initial analysis of 15 pooled ears revealed that, at 5 dpi, the transcripts of all five *TRI *genes were detected at their highest level at the active hyphal front of infection and decreased as the distance from the advancing front of infection increased (Figure 5(a)). All the *TRI *genes showed a greater than twofold change in abundance in RI-3 (symptomless infection front) compared to RI-1 (the onset of disease symptoms, origin of rachis infection). *TRI5 *expression was very low in RI-1 and exhibited a dramatic 11.90-fold increase in abundance between the RI-1 and the advancing hyphal front in the RI-3 tissue, reaching a modest expression level (Table 2). *TRI4*,* TRI9*, and *TRI14 *expression was substantially higher than *TRI5* in RI-1, whilst their increase in expression level was more modest in RI-3. Interestingly, the relative level of expression of *TRI6* was considerably lower than the four other *TRI *genes, namely, *TRI4*, *TRI5*, *TRI9*, and *TRI14*, throughout all rachis internodes.

The later 7 dpi time point was studied in more detail and included 45 ears (3 pools of 15 ears). This time point was chosen to determine whether elevated *TRI *gene expression at the advancing front of infection was retained throughout the progression of infection. At 7 dpi, the first two rachis internodes (RI-1 and RI-2) represented fully symptomatic infection, the following two (RI-3 and RI-4) harbour the onset of disease symptoms, while the final two colonised internodes (RI-5 and RI-6) represented symptomless infection. The *TRI *genes that had shown the highest level and the greatest change in expression, at 5 dpi, were chosen for further analysis. The expression of *TRI4 *and *TRI5*, relative to fungal biomass, again was higher during symptomless infection (Table 2). Within the fully symptomatic plant tissue, *TRI *gene expression was very low. The amount of fungal biomass demonstrated an inverse relationship to *TRI *gene expression (Figure 5(b); Supplementary Figure 5).

## 3. Discussion

### 3.1. The Effectiveness of the Reporter Strain to Track *Fusarium graminearum* Infection

Our objective was to create a *F. graminearum* reporter strain that would simplify the quantification of the progression of infection throughout the ear. The GUS reporter strain had wild-type growth rates, pathogenicity, and mycotoxin production. *In planta *experiments confirmed that the constitutive GUS expressing strain can be used to quantify fungal biomass within both the symptomatic- and symptomless-infected wheat tissue. Histochemical and biochemical analyses verified the presence of a substantial phase of symptomless growth ahead of disease symptoms, as previously noted by Brown et al. [12]. Therefore, in this interaction, there is a considerable latent period between the tissue becoming infected and the development of macroscopically visible symptoms. These studies also showed that a spikelet(s) at the tip of the ear and in other locations can fail to become infected even though the ear is otherwise heavily diseased. The histochemical and microscopy analyses of sectioned tissues confirmed that GUS activity was detected within the *Fusarium* hyphae. Analysis of the *Fusarium* infection process using the constitutive GUS expressing strain revealed a considerable reduction in GUS activity at late time points in infection, which is believed to be caused, in part, by the reduction in physiologically active hyphae. It is currently unknown what triggers the increase in abundance of “ghost” hyphae in the later stages of infection of a susceptible wheat genotype. In various other host-pathogen interactions, when compatible interactions have been quantitatively analysed using fungal transformants that contained the *gpdA*:*GUS *reporter construct, a steady increase in the levels of detectable MUG activity over the time-course has usually been reported. For example, in the interaction between tomato leaves and the intercellularly growing biotroph *Cladosporium fulvum* (recently renamed *Passalora fulva*), a 100-fold increase in detectable MUG activity was observed over the 12-day time course prior to the onset of sporulation [23]. The final developmental stage in the *Fusarium*-wheat ear infection process is sporulation and the redistribution of resources to areas of sporulation may concentrate activity to these regions of the colony. 

In the *Fusarium-*wheat floral interaction, several major quantitative trait loci (QTL) conferring resistance have been identified [24]. Using this new *F. graminearum* GUS reporter strain and the now available near-isogenic germplasm, it should be possible to evaluate, quantitatively, the kinetics of biomass accumulation associated with the presence/absence of each QTL in different wheat backgrounds. This should reveal whether the various genetic sources of either type 1 or type 2 resistance differentially influence the rate of loss of viable *Fusarium *hyphae. The constitutive GUS expressing transformant could be particularly informative in this regard because its use should simplify the identification of the onset of the formation of “ghost” hyphae and/or reductions in fungal biomass conferred by the different QTLs when present in various wheat backgrounds. Also, the ability to stain entire wheat ears and then to slice vertically to visualise the entire internal infection process using light microscopy (Figure 1) will greatly assist the evaluation of wheat germplasm for the extent of symptomless infection compared to the macroscopically visible disease symptoms present. This relatively rapid procedure could be used to eliminate lines which supported predominantly symptomless infections. Overall, the GUS reporter strain provides a higher throughput system to accurately quantify physiologically active infection by easy histochemical and biochemical means, compared to the alternative fluorescent reporter proteins which require the use of confocal microscopy and a higher level of training.

### 3.2. Mycotoxin Production during the Early Phases of Infection

The early infection process was explored further using the simplified linear rachis internode system and RT-qPCR to determine the expression of various *Fusarium *genes required for mycotoxin production. *TRI *gene expression was found to be maximal at the hyphal front at both 5 and 7 days post spikelet inoculation. This indicates that the mycotoxin induction pattern is maintained as the hyphal advance symptomlessly through newly colonised wheat tissue. In addition to the upregulation of trichothecene biosynthetic genes (*TRI4*,* TRI5*, and *TRI9*), these quantitative analyses also revealed the elevated transcription of *TRI* gene regulators *TRI6 *and *TRI14*, at the advancing front of infection. Deletion of the *TRI6 *gene, which codes for a transcription factor, downregulates more than 200 genes, including most *TRI *genes and many genes from the preceding isoprenoid pathway, resulting in a reduction in DON production and pathogenicity [22]. Interestingly, a *F. graminearum TRI14 *deletion strain demonstrated wild-type DON production *in vitro* while losing the ability to produce DON *in planta* resulting in a reduction in pathogenicity [21]. The combination of these results implies that *TRI6 *and *TRI14* regulation of the *TRI *genes is essential for DON production and pathogenesis during establishment of symptomless wheat ear infections. Expression of all of the *TRI *genes was minimal in the symptomatic wheat tissue within which an abundance of *Fusarium *hyphae are associated with dead plant cells. This suggests that, for this phase of the infection process, DON production is either not required or the tissue already contains considerable DON because of the earlier colonisation. 

The mycotoxin, DON, and its acetylated derivatives, produced by *F. graminearum* during infection of susceptible wheat, have been demonstrated to be essential for the spread of infection beyond the inoculated spikelet [15]. For wheat cultivar Bobwhite and the sequenced *F. graminearum *strain PH-1 [25], this result has been verified [16]. In the absence of DON production, symptomless infection is lost and the wheat plant is able to lay down a defensive barrier which appears as a brown ring around the lesion [16]. The production of DON may therefore inhibit the defensive response of plant cells in the vicinity of invading hyphae, minimising the onset of various cell wall reinforcement processes [9] by preventing protein synthesis [17]. This strategy would assist *Fusarium* infection. Indeed, previous detailed microscopic analyses revealed minimal changes to the appearance of wheat cortical cells surrounding the advancing DON-producing *Fusarium* hyphae [12]. For DON production to be relatively uniform throughout the plant tissue at an early stage in the infection, the limited number of intercellular hyphae at the actively advancing front of symptomless infection would need to produce a higher amount of DON per fungal cell compared to fungal cells further into the colony where the ratio of fungal cells to live plant cells is consistently higher. Accordingly, once the plant cells have died, DON production would no longer be required.

## 4. Experimental Procedures

### 4.1. Fungal Strains, Media, and Culture

The *F. graminearum *wild-type strain PH-1 was used throughout this investigation. The fungus was routinely cultured on SNA (synthetic nutrient poor agar) plates containing 0.1% KH_2_PO_4_, 0.1% KNO_3_, 0.1% MgSO_4_  ×  7H_2_O, 0.05% KCl, 0.02% glucose, 0.02% sucrose, and 2% agar. Plates were incubated at room temperature and under constant illumination from one near-UV tube (Phillips TLD 36W/08) and one white light tube (Phillips TLD 36W/830HF). Soil stocks were stored at −80°C. To remove old conidia and induce fresh conidia formation, 10-day old SNA plates were washed with an overlay of TB3 (0.3% yeast extract, 0.3% Bacto Peptone, and 20% sucrose). After 24 h, a conidial suspension was harvested in sterile water, filtered through miracloth (Calbiochem), and then adjusted to a concentration of 4 × 10^4^/mL with sterile water.

### 4.2. Wheat Cultivar, Growth of Plants, and Plant Inoculations

The susceptible wheat (*Triticum aestivum*) cultivar, Bobwhite, was used as the host plant throughout this investigation. Plants were grown in a glasshouse as previously described [26]. At the first appearance of anther extrusion, 5 *μ*L of a 4 × 10^4^/mL conidial suspension was placed in the floral cavity between the palea and lemma of the outer two florets in the middle of the ear. Control plants were inoculated with sterile water only. Inoculated plants were incubated in a humid chamber for 72 h of which the first 24 h were in darkness. The inoculated plants were then kept in the glasshouse at ambient humidity.

### 4.3. Production of the *F. graminearum* Constitutive GUS Reporter Strain

Two plasmids were used to create a transgenic strain of *F. graminearum* strain PH-1 which constitutively expresses the GUS enzyme. The first, pNOM102, harbours the GUS gene from *Escherichia coli* (*uidA*) under the control of the *gpdA* promoter from *Aspergillus nidulans *[19]. The second, pHyg1.4, contains the hygromycin phosphotransferase gene (*hph*) under the control of the *trpC* promoter. The pHyg1.4 plasmid was created by ligating a 1.4 kb *Hpa*I fragment from pCB1004 [27] into pBluescript II-SK+ (Stratagene) cut with *Eco*RV. Plasmids pNOM102 and pHyg1.4 were linearised with *Hin*DIII and *Bam*HI, respectively, before being cotransformed into PH-1 protoplasts following the protocol described by Urban et al. [28]. Fungal transformants were subjected to two rounds of selection on minimal media plates (SNA) containing hygromycin (75 *μ*g/mL) and then single spored.

Stable GUS expressing transformants were checked for wild-type growth rates on a minimal and nutrient rich agar (SNA and PDB) and for asexual and sexual spore production (conidia and perithecia) by growth under a mixture of near UV and white light on either SNA or carrot agar as previously described [26, 29]. Wheat ear infection assays were used to assay the virulence of transformants and the wild-type PH-1 progenitor strain [28]. The level of DON production of selected transformants was compared to the parent strain PH-1 by growing triplicate plates of each strain on corn meal agar (20 g/L maize flour, 20 g/L agar) for 14 days at high humidity in an incubator at 28°C. The deoxynivalenol (DON) concentration was then quantified using the RIDASCREEN FAST DON ELISA kit from r-biopharm following the manufacturer's protocol. The selected transformant was G3.

### 4.4. Evaluation of Fungal Biomass via X-gluc Histochemical Staining and MUG Fluorometric Assay

Wheat ears infected with the constitutively GUS expressing strain were dissected longitudinally with a scalpel and submerged in a 50 mM Na_2_HPO_4_/NaH_2_PO_4_ buffer (pH 7) solution containing 0.5 mg/mL X-gluc (5-bromo-4-chloro-3-indolyl-beta-D-glucuronic acid, cyclohexylammonium) (Sigma, UK), 0.05% Triton X-100, 1 mM K_3_[Fe(CN)_6_], and 1 mM K_4_[Fe(CN)_6_] · 3H_2_O. Submerged ears were vacuum infiltrated for 1 h at room temperature and then incubated at 37°C for 4 h. Stained ears were cleared in 70% ethanol [30].

Three wheat ears infected with the constitutive GUS expressing or the wild-type strain were scored for disease symptoms and then dissected. Individual spikelets were ground with steel beads in 500 mL GUS extraction buffer (50 mM NaHPO_4_ (pH 7), 10 mM dithiothreitol, 1 mM EDTA (pH 8), 0.1% sodium lauryl sarcosine, 0.1% Triton X-100) using a Retsch Mixer Mill 200. The supernatant was collected after centrifugation (5 min at 10,000 rpm); subsequently, 50 *μ*L was used in the MUG assay as described by Jefferson [30].

### 4.5. Preparation of Individual Tissues of the Wheat Ear for Light Microscopy

Selected inoculated wheat ears were photographed before sampling. Successive rachis internodes below the point of inoculation were individually imaged on a Leica stereomicroscope prior to being excised. Tissue samples were individually fixed for 16 h with 4% paraformaldehyde (Sigma) 2.5% glutaraldehyde (Sigma) in 0.1 M Sorensen's phosphate buffer (NaH_2_PO_4_ : Na_2_HPO_4_, pH 7.2) then washed 3x with 0.05 M Sorensen's buffer and once with sterile water. Samples were subsequently dehydrated in a graded ethanol series, embedded in hard grade LR White (TAAB), and polymerised at 60°C for 24 h [31]. 

Transverse semi-thin 1 *μ*m sections were cut with a glass knife on an ultramicrotome (Reichert-Jung, Ultracut). Sections were collected on glass slides and dried on a hot plate set at 40°C. After staining with aqueous 0.1% toluidine blue O (TBO) in 1% sodium tetraborate pH 9, sections were mounted in DPX (Sigma), then examined and imaged using a Zeiss Axiophot light microscope. The images presented in this study are representative of the three biological replicates carried out for each experiment.

### 4.6. Extraction of RNA from Rachis Internodes

The four or seven rachis internodes (relative to the dpi when harvested) below the inoculated spikelet of each ear were individually excised and frozen in liquid nitrogen at 5 dpi and 7 dpi. Following visual inspection, in total 15 representative wheat ears were dissected for each treatment, mock-inoculated or *F. graminearum*-infected, and the internode segments collected. Freeze-dried samples were ground in liquid nitrogen with a pestle and mortar. Total RNA was extracted using the TRIzol reagent (Invitrogen) with minor modifications to manufacturer's instructions. Total RNA was precipitated at −20°C for 16 h with 8 M lithium chloride. Purified RNA was quantified by absorbance at 260 nm.

### 4.7. Quantitative RT-qPCR Gene Expression Analysis

Total RNA was treated with a DNA-*free* ^TM^ kit (Ambion) to remove contaminating DNA and the absence of genomic DNA confirmed by PCR using intergenic primers. The cDNA was synthesised from 1 *μ*g of total RNA using 500 ng of Oligo-dT primers and 200 U Superscript III (Invitrogen) according to manufacturer's instructions. RT-qPCR was performed on a Real-Time PCR System 7500 (Applied Biosystems). The sense and antisense primers used are presented in Supplementary Table 1. A standard curve of gDNA with known concentrations ranging from 25 ng to 0.025 ng was created for each primer pair. Synthesised cDNA was diluted 1/50 in sterile H_2_O. The PCR analysis was performed in a final volume of 20 *μ*L and consisted of 10 *μ*L of Jumpstart Taq Ready mix plus ROX (Invitrogen), 5 *μ*L of 1.2 *μ*M of each primer, and 5 *μ*L of the relevant nucleic acid template. Thermal cycling conditions were as follows: [95°C 2 min, (95°C 15 sec, 62°C 20 sec, 72°C 45 sec) × 40]. The dissociation curves were as follows: [95°C 15 sec, 60°C 1 min, 95°C 15 sec] × 1. The average cycle threshold value for each sample was calculated from triplicate technical replicates. Fungal housekeeping genes *γ*-actin and *β*-tubulin were used to normalise the RT-qPCR data for fungal biomass.

## Supplementary Material

Supplementary Figure 1: Confirmation that X-gluc specifically stains the hyphae of constitutive GUS expressing strain.
Supplementary Figure 2: The identification of fungal infection in the first three rachis internodes at 5 dpi.
Supplementary Figure 3: The extracted RNA from the radius internodes of the PH-1 infected and the water-only control (Mock) at 5 dpi.
Supplementary Figure 4: The extracted RNA from the rachis internodes of the PH-1 infected and the water-only control (Mock) at 5 dpi is free of genornic DNA (gDNA) contamination.
Supplementary Figure 5: Fungal *TRIO* (A) and *TRI5 (B)* gene expression in the infected rachis internodes in which *F*. * graminearum* was detected at 7 dpi.
Supplementary Table 1: Fungal genes selected for expression analysis by RT-qPCR, their FGSG locus ID, BROAD (http://www.broadinstitute.org/)/MIPS (http://www.mips.helmholtzmuenchen.de/) function and their primer sequence.

## Figures and Tables

**Table 1:** GUS activity within the individual wheat spikelets at 8 and 16 dpi, as determined by the MUG assay, demonstrates a dramatic drop in MUG activity and therefore active fungal biomass at 16 dpi. In technical replicates, the values obtained never varied more than 5% from each other. This data is from a representative ear, from each time point, of the six explored in detail.

Spikelet	MUG (nmol/min)	
	8 dpi	16 dpi
1	8.8	8.9
2	0.0	0.0
3	43.2	10.0
4	1397.5	41.7
5	1517.4	47.9
6	1863.3	51.6
7	1732.7	63.1
8	129.4	44.3
9	0.0	34.1
10	12.5	30.5
11	0.0	34.3
12	0.0	0.0
13	0.0	34.9
14	0.0	25.5
15	0.0	9.1

Sum	6704.8	435.8
*Fold drop in MUG activity*		**15.3847**

**Table 2:** Fold change in fungal gene expression between the symptomless (RI-3) and the microscopically symptomatic (RI-1) at 5 dpi. Fold change in fungal gene expression between the symptomless (RI-6) and the microscopically symptomatic (RI-1) at 7 dpi. Each gene showed a greater than twofold change in expression. Standard errors for the three biological replicates of the more in depth analysis, at the 7 dpi, are given in brackets.

Fungal gene	Fold change in gene expression	
	5 dpi	7 dpi
*FgTri4*	5.14	5.92 (0.32)
*FgTri5*	11.90	6.03 (0.49)
*FgTri6*	4.31	—
*FgTri9*	2.68	—
*FgTri14*	5.96	—
