# Supplementary material for: Characterisation of the Fusarium graminearum-Wheat Floral Interaction
Source: J Pathog. 2011 Oct 5;2011:626345. doi: 10.4061/2011/626345 (PMC3335584; doi:10.4061/2011/626345)
Supplement: Supplementary file 1 — Supplementary Figure 1: Confirmation that X-gluc specifically stains the hyphae of constitutive GUS expressing strain. Supplementary Figure 2: The identification of fungal infection in the first three rachis internodes at 5 dpi. Supplementary Figure 3: The extracted RNA from the radius internodes of the PH-1 infected and the water-only control (Mock) at 5 dpi. Supplementary Figure 4: The extracted RNA from the rachis internodes of the PH-1 infected and the water-only control (Mock) at 5 dpi is free of genornic DNA (gDNA) contamination. Supplementary Figure 5: Fungal TRIO (A) and TRI5 (B) gene expression in the infected rachis internodes in which F. graminearum was detected at 7 dpi. Supplementary Table 1: Fungal genes selected for expression analysis by RT-qPCR, their FGSG locus ID, BROAD (http://www.broadinstitute.org/)/MIPS (http://www.mips.helmholtzmuenchen.de/) function and their primer sequence. [file 626345.f1.pdf]

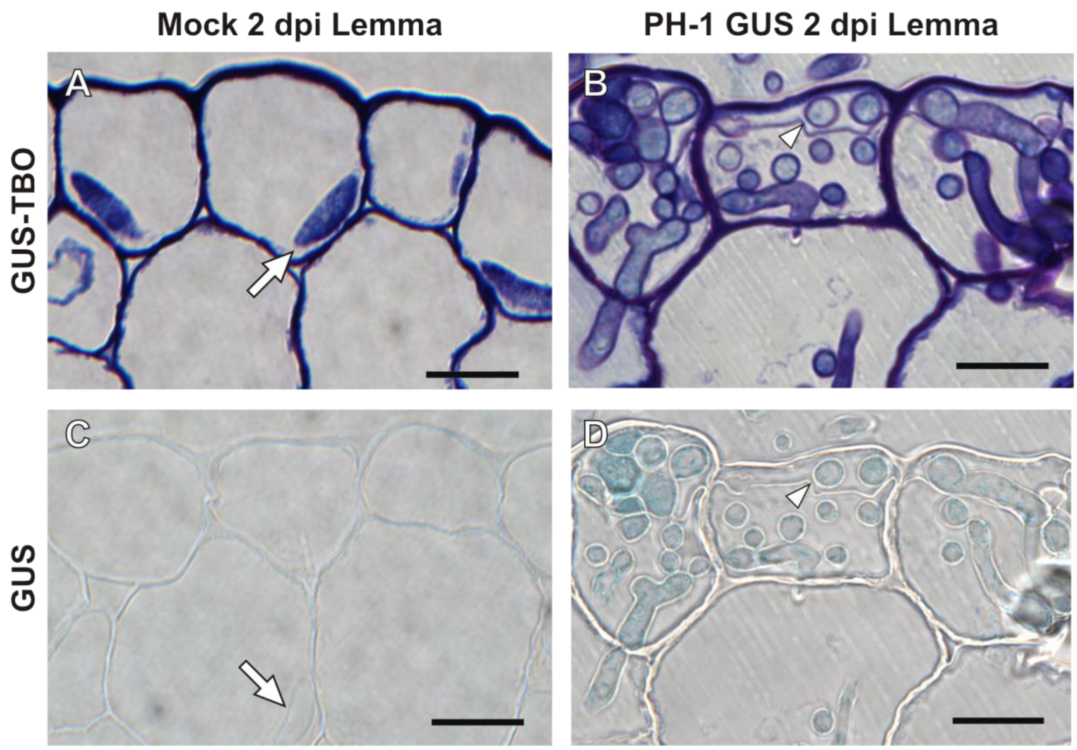

Supplementary Figure 1 Confirmation that X-gluc specifically stains the hyphae of constitutive GUS expressing strain. All images in this figure are of the lemma from the inoculated floret at 2 dpi, which was stained with X-gluc prior to fixation. All images are of sequential transverse 3 µm LR white sections; panels (A) and (B) are stained with 0.1 % TBO, pH 9, while panels (C) and (D) are not. White arrow = host nuclei, white arrowhead = intercellular fungal hyphae. Bar = 15 µm.

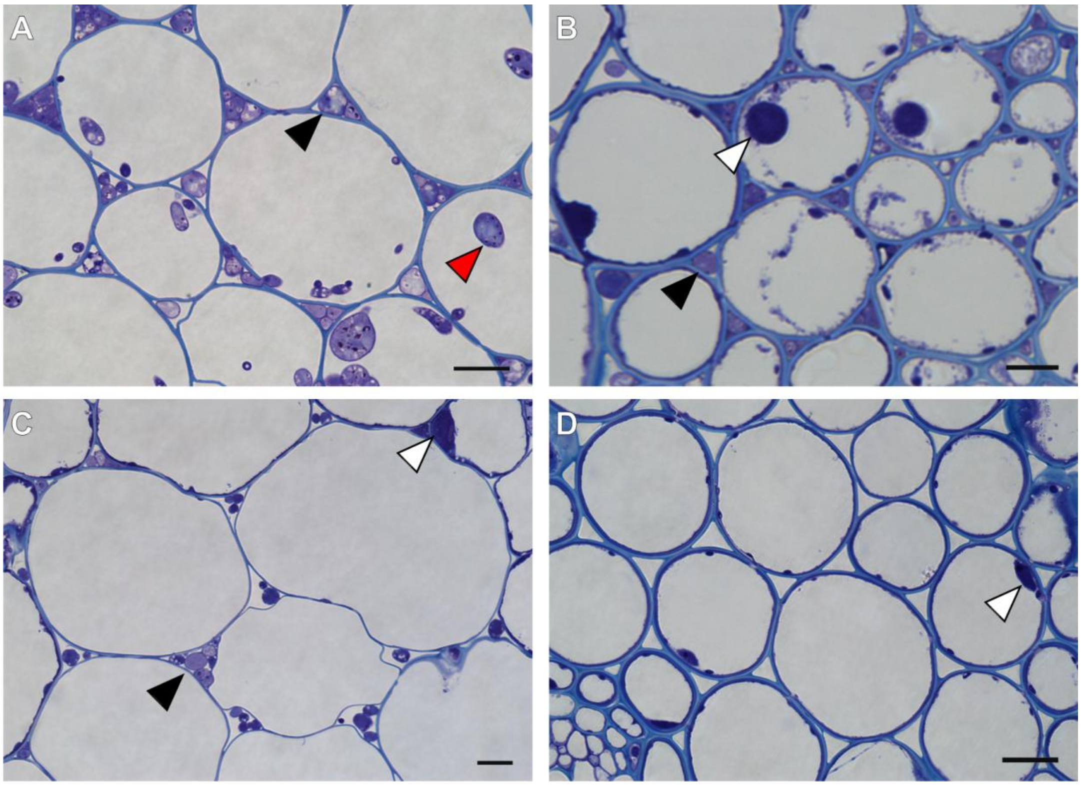

Supplementary Figure 2 The identification of fungal infection in the first three rachis internodes at 5 dpi. Images of transverse 1 µm LR white sections of cortical cells within various rachis internodes stained with 0.1% TBO pH 9. The first (A), second (B), third (C) and fourth (D) rachis internode below the point of inoculation at 5 dpi. Generic legend: black arrowhead = intercellular hyphae, red arrowhead = intracellular hyphae, white arrowhead = host nuclei. Bar = 15 µm.

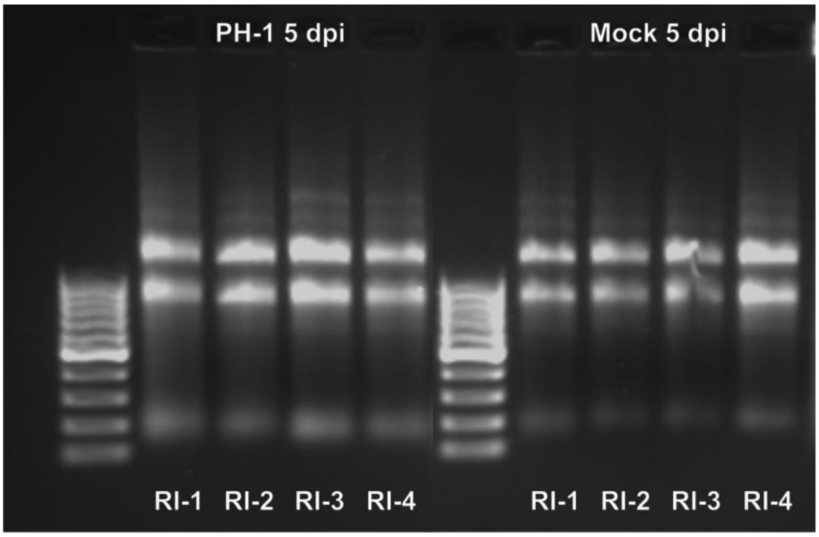

Supplementary Figure 3 The extracted RNA from the rachis internodes of the PH-1 infected and the water-only control (Mock) at 5 dpi. One µg of total RNA was separated on a 1 % agarose gel alongside a 100 bp DNA ladder (Generuler, Fermentas). Legend: RI-1 to RI-4 = the sequential rachis internodes below the inoculated spikelet.

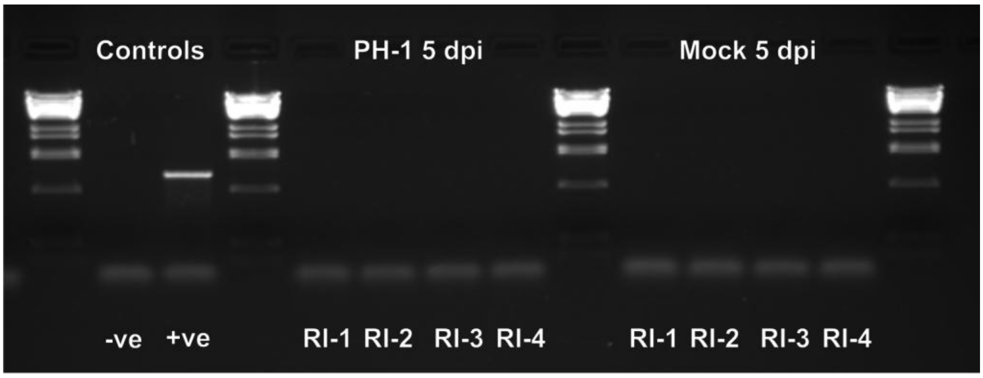

Supplementary Figure 4 The extracted RNA from the rachis internodes of the PH-1 infected and the water-only control (Mock) at 5 dpi is free of genomic DNA (gDNA) contamination. Intergenic primers that only amplify gDNA were used. The presence of an amplicon (902 bp) on the 1 % agarose gel, separated alongside a lambda_DNA_BstE II ladder, represents the presence of gDNA. Legend: RI-1 to RI-4 = the sequential rachis internodes below the inoculated spikelet, –ve = the non template negative control, +ve = the gDNA positive control.

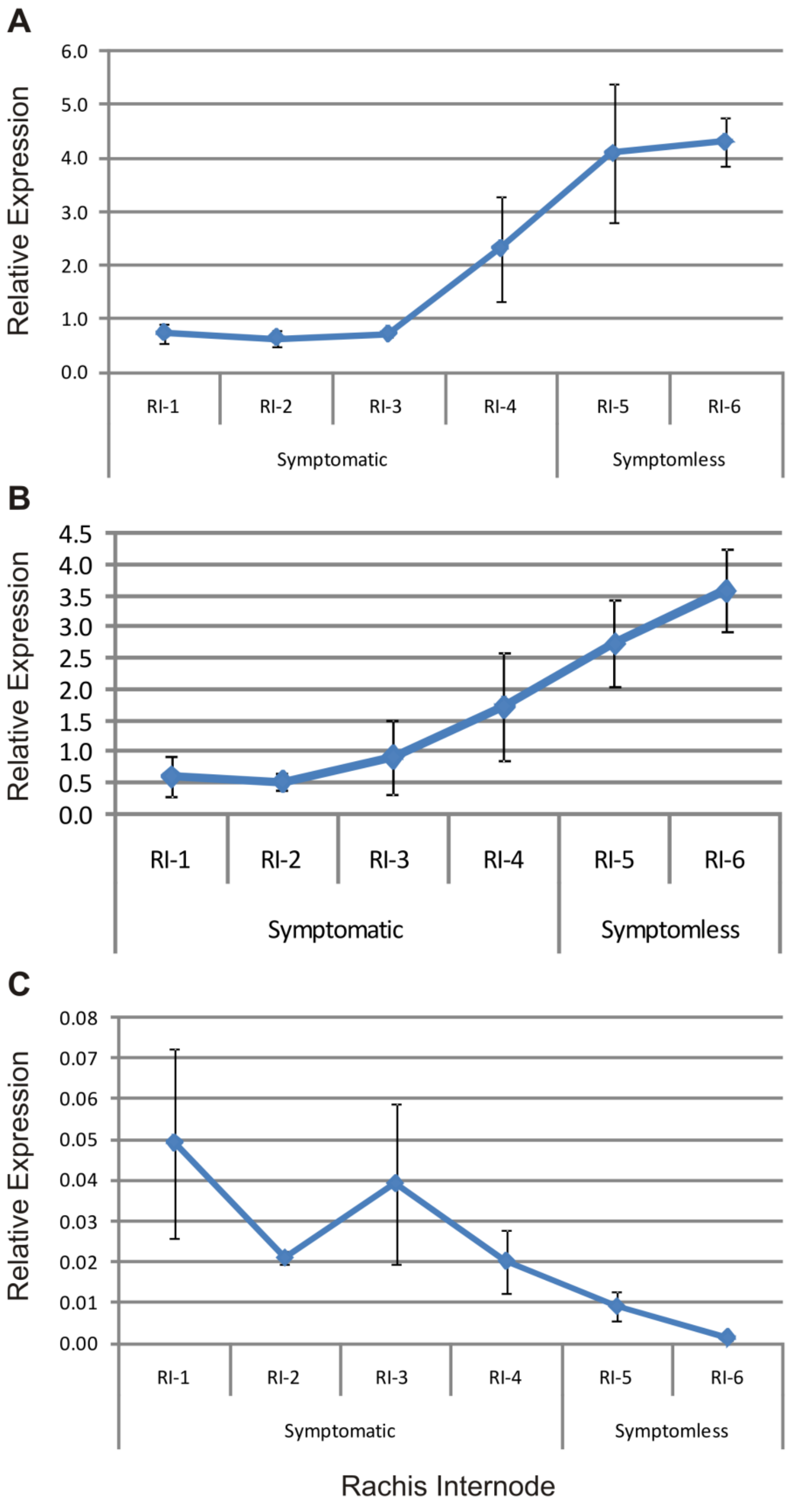

Supplementary Figure 5 Fungal *TRI4* (A) and *TRI5* (B) gene expression in the infected rachis internodes in which *F. graminearum* was detected at 7 dpi. Relative expression was measured by RT-qPCR and values normalised for fungal biomass relative to the expression of *F. graminearum* γ-actin and β-tubulin (C). Biological error is represented by one standard deviation from the mean.

Supplementary Table 1 Fungal genes selected for expression analysis by RT-qPCR, their FGSG locus ID, BROAD (www.broadinstitute.org) / MIPS (www.mips.helmholtz-muenchen.de) function and their primer sequence.

Fungal gene	FGSG locus ID	Broad / MIPS function	Primer sequence (5'-3')	
			Sense	Antisense
FgActin	07335.3	Actin / Probable Actin	ATGGTGTCACTCACGTTGTCC	CAGTGGTGGAGAAGGTGTAACC
FgTubulin	09530.3	Tubulin beta chain / Beta-tubulin	TCAACATGGTGCCCTTCC	TTGGGGTCGAACATCTGC
FgTri4	03535.3	Trichodiene oxygenase / Trichodiene oxygenase [cytochrome P450]	AGACTACTTCAAGGACACTGGCC	GGTAAGGGAGATTCTCTAGGGTAGC
FgTri5	03537.3	Trichodiene synthase [sesquiterpene cyclase] / Trichodiene synthase	GATGAGCGGAAGCATTTCC	CGCTCATCGTCGAATTCC
FgTri6	03536.3	Hypothetical protein similar to regulatory protein / Trichothecene biosynthesis positive transcription factor (TRI6)	TGTCGCTACTCAGAATGCC	CCCTGCTAAAGACCCTCA
FgTri9	03539.3	Conserved hypothetical protein / Hypothetical protein	TATCCACTCAAACACTCACCCC	TGGTAGCGCATAAAGCAGC
FgTri14	03543.3	Hypothetical protein similar to TRI14 / Putative trichothecene biosynthesis gene	CTGATAAGCTTGAACCACCTCG	TTGATCACAACGGGAGTTCC
